# The Dentine, Cellular and Fibrous

**Published:** 1873-06

**Authors:** Geo. B. Harriman


### DENTAL REGISTER.

VOL. XXVII.] JUNE, 1873. [No. 6.

### THE DENTINE, CELLULAR AND FIBROUS.

BY PROF. GEO. B. HARRIMAN.

The above is the heading of an article in the October number of the DENTAL REGISTER, 72, on which I propose in this paper to make some comments, by the author's permission, (S. P. Cutter, M. D., D. D. S.,) Memphis, Tenn., already granted in the article.

On page 407 I find the following : "As has been stated in previous articles, erroneous terms are employed by writers, when considering the structure of the teeth. Because such terms lead to dangerous practice, they demand further attention."

Year after year we read of "tubes," "tubuli," prisms, hollow pipes, etc. It seems that the writer differs with those who claim that the dentine has tubes, or is tubulated. On the contrary, he does not believe in or admit the existence of any such structure in the dentine ; he thinks they are constructed of *cells and fibres*, and thinks that our former notion leads to dangerous practice.

Now, if such be actually the facts in the case, I think it high time the profession was set right. That is just what is needed at this day and time, as *we* claim as a profession to be very *learned, scientific, and wise*, in all our knowledge bearing in any way on theoretical or practical points.

I quite agree with the writer in the terms fibres and cells, and really have long since given into the idea, that all, or say nearly all organic structures, are made up of cells in some form, and many tissues, as nerves, muscles, and other tissues, are composed of fibres formed from cells, the latter being first formed, and when marshalled into line become fibrous, whether in single file, as in dentine, or solid phalanx, as in muscular tissues and other structures ; still somehow or other I still cling to the idea that dentine has tubes, and these tubes are occupied by true nerve-fibres or fibrils, and that those fibrils are composed of single file cells in linear arrangement, forming, what the writer terms, axis cylinder of nerve fibre. Here we do not disagree at all, only all arrive at the same conclusion in a different way.

In relation to the true structure of the solid dentine we do somewhat differ, as I still believe that the solid dentine has tubes, and those tubes have solid walls, and those tubes are filled with soft animal fibres, which I believe to be true nerve fibres, with axis cylinder of sensorial nutrient functions, of the highest endowment, because having to resist all the shocks, the consequence of outcrossing of hard bony structures, exposed as they are to heat, cold, moisture, dryness, friction wear, concussion, and disease, which no other tissues of the entire body are subjected to.

The writer speaking of a tooth sent to him, by T. W. Clements, D. D., Pembroke, Me., says, same page, "The tooth had been extracted five years at least before the section was made, and it represents the cells and fibres most perfectly, and demonstrates beyond a doubt, that the soft solid substance has not dried up."

I can not exactly give in to the idea of the soft solid substance in the tubuli remaining soft all this time, as that seems to me to be contrary to physical laws governing universal structure.

We know that when the pulp in a tooth dies, or is killed, and the cavity allowed to remain unstopped for a considerable length of time, the tooth, as a rule, turns dark, and we have generally supposed that dark color was caused by the absorption into the tubuli, or pores, as some would term them, of carbonaceous matters, or some principle contained in the juices of the mouth ; and if the soft fibre still remained intact, no such discoloration could take place at all.

We might account for this discoloration from the decomposition of the soft fibre, in part at least ; still the amount of decomposition of so trifling an amount of animal matter could not amount to much, and we must conclude that a certain amount of absorption into the tubes must take place, and there decomposing, causing the discoloration ; in no other way can we attempt to account for this change in color.

Now if the fibres decompose or evaporate in any way from the dentinal tubes in the mouth, the same state or similar state of things, would take place out of the mouth, and the tubes would become emptied in some way of their organic occupants.

Put a tooth into the fire, and witness the result : smoke passes out from the tubuli if there be a cavity in it, if not through foramen of fang, and continues to escape until all the animal portion *so called* escapes, and the tooth has lost in weight in proportion to the animal to the mineral *so called* matter of the teeth ; caustic alkalies also take out the animal matters. In both cases the tubular structure remains intact, or nearly so, and presents just about the same appearance of a tooth not so treated, so far as I have been able to determine under the microscope.

On the other hand, when the lime has been all removed from dentine by acids or decay, specimens of such dentine also retain the tubular structure, sometimes intact, and in their normal appearance ; still the dentine has lost in weight, just in proportion to the amount of lime so removed. Now all this lime has to be washed out through these tubes we are discussing. In both instances of displacement, there remains still the ghastly structure of dentine, nearly or quite normal in appearance.

To my mind these conclusions are satisfactory as regards the tubulated structure of dentine. Still I agree with the author in the main, that is, that the tooth is made up of cells and fibres ; the entire solid dentine, or the intertubular spaces are made up of a cell structure, and the tubular occupancy composed of fibres, which I have long maintained, are genuine nerve fibres, before any writer had ever positively asserted the same thing, which reference to some of my former papers will show. In this respect I took strong grounds against great opposition.

I have never used anything like as high powers as the above author in my investigations ; from the fact that I could not get light enough to give satisfaction. I used mostly 500 diameters, sometimes 700, giving a power not more than from 250,000 to 490,000 surfaces, which would magnify the tubuli from a quarter to half a million of times, with a good light, and good defining power.

In using a power of from 5,000 to 10,000 diameters, the specimen would be magnified from twenty-five to one hundred millions of surfaces, or power, a powers so enormous, that but very faint light at least could be had, and a cross section of dentine would show openings large enough to run the finger through, seen by a dim shadow only, no matter how the light may be condensed. On page 408 I shall have to *break a lance* with the author on several points.

Speaking of anastomosis of cells and fibres, *nerves*, *tubuli*, at junction of enamel and dentine, I must beg to differ very widely from him. In the cut on same page the *tubuli*, *nerves*, *cells*, *and fibres*, are represented as several of them uniting just before reaching enamel. Right here I take positive issue, and boldly state, without fear of contradiction, that no such anastomosing ever exists at or near the enamel, or any where else in the dentine of the crown of the tooth, where the tubes or fibres come up to enamel.

On the very contrary, every solitary tube that reaches the enamel, and all do so reach that start for it, bifurcate or fork, at least once, and sometimes trifork just before reaching the enamel, without any exception in what has been designated the coronal region of branching, in normal, or sound and healthy dentine.

The cut represents the tubes running with the enamel, in some places, and somewhat enlarged. This fact I have noticed in some specimens, at certain points, generally over the crown under the cusps, at which point, such teeth are generally sensitive to the file as it approaches the dentine, speaking theoretically, not sufficiently demonstrated as yet, owing to limited observation in this connection. Neither did I ever find any enlargement of tubuli until the enamel is reached, and as I have often stated, each and every tubuli has a distinct and bold terminus *per se* at the enamel, and no circuits or anastomosis so far as my observations have extended, notwithstanding there are many authors who may, and do differ with me on this point.

So far as seeing through the holes in a cross section or not, we do see what to me appears to be openings, so much so that light passes through, or apparently so, under 500 diameters, very distinctly.

The cuts on page 409 in fact, represent the same thing *in* an opaque substance with apparent perforation, though *microscopic*, as in all other minute organic structures ; they are all microscopic, and cannot be seen with the naked eye.

Same page, writer says, in the so-called "tubuli" of the dentine, we not only find cells and fibres of connective tissue, but in many instances nerve fibres and ganglion cells can be traced from the pulp cavity to the junction of the enamel, dentine, and cementum.

I must confess I have never seen any difference in any respect between the contents of one tube and any other. The nerve fibre I adopt as a fact, but as to the ganglion cells in the tubuli I must acknowledge I have never seen them, unless the beaded, or baccuted appearances, to be recognized in all tubuli, more or less distinct, may be regarded as such.

This beaded appearance to my judgment might be imagined to correspond to cell structure throughout the system of tubuli.

As to connective tissue fibres being found in the tubuli, I have no good reason to believe they exist, notwithstanding the writer's views, also those of Francis Ball, of Germany, whose paper I criticised a year or two since, on the same subject. Now, as then, I must state that I can see no use nature can make of connective tissue fibres in the tubuli, unless she wished to make an idle display of unnecessary complications where no useful object could be obtained.

Cut on page 410 represents terminal fibres at junction of enamel in a ganglion. I am still clearly of the opinion that the writer has in some way been misled in this junction by his chemical process in preparing his specimen, for I am quite sure he could not see them in dentine unacted on by chemical agents.

There is a region, which I have on one occasion designated as *terra incognita*, or unknown land situated between the enamel and dentine, of a granular, undefined strata, just where the tubuli terminate, though generally passing, this region square up to base of enamel, which may be distinctly seen in properly prepared specimens of healthy formed teeth, if not all teeth in fact.

Same page, speaking of the medullary substance, I shall not attempt to discuss, not having given the subject sufficient special attention to do so, presuming at the same time that the writer may be correct in his views on that subject, having received but little attention from other writers.

On page 411 he says, speaking of the terminations of nerves, "If we take the teeth here, we find the nerve fibre passing through into the dentine, towards the enamel and cementum, and in some instances they can be traced entering its substance. As the fibres approach their termination, they draw together, and unite by anastomosis, or rather they enter in a cell or ganglion, but in no instance do they terminate by free extremity." "This formation somewhat confirms the position that all nerve fibres form a complete circuit, for the current of nerve force, whether it be motor or sensor. I am confident they do not end in points or filaments; nerves in teeth have no ends, except as they are continuous with the terminating of another nerve fibre, or as they terminate in what I will call a ganglion cell."

"This termination of nerve fibre in dentine and enamel, this ganglion or cell termination of nerves in teeth, never before demonstrated, now being proved and accepted, we have now to seek in some form for the cause of excessive sensation in teeth."

Speaking of the fibres of nerves in teeth ending by anastomosis, and that the fact was now proved and demonstrated, and accepted, I must beg to differ widely from the writer, although the idea of anastomosis has been, and is still believed by many able investigators. As before stated, I have satisfactorily, to myself, demonstrated to the contrary which I have already explained. Let observers carefully examine for themselves. We are all, I believe, agreed that only one set of nerve fibres supply the teeth, and as the nerve currents travel in opposite directions, in the opposite sets of nerves we must conclude that the nerves of the teeth, at least, if not others, have no anastomosing circuits, unless we admit the fact that a nerve of the same denomination carries on a double current, which to me seems impossible, as only a sensorial current is carried from the teeth to the brain, and no return or reflex current that we know any thing about. There is no need of any other current. It would be more consistent to imagine a circuit current through the two sets of nerves still the fact remains to be proven, that any such current is sustained by anastomosis of nerve filaments. We know that ligating or separating any nerve, a complete arrestation of function, through such nerve, at once takes place, without in any way interfering with any other nerve function, that might be supposed to anastomose with such divided nerve.

If the nerve function depend upon a complete circuit by anastomosis, the severing of either the efferent or afferent branch, would necessarily so disturb the circuit current as to arrest function in both.

There are cases of paralysis of one set of nerves, and not the other set, running to a part more or less complete; in other instances both sets are more or lesscompletely paralyzed.

These facts to me rather favor the independent nerve fibrilar termination, than that of circuits through anastomosis; at the same time we must admit the fact of a magnetic conducting intermediate *sarcode* everywhere in the body, which may serve as a *vinculum* between the two sets of nerves.

